# The role of doulas in providing breastfeeding support during the COVID-19 pandemic

**DOI:** 10.1186/s13006-023-00558-0

**Published:** 2023-04-21

**Authors:** Monica Ochapa, Kesha Baptiste-Roberts, Sharon E. Barrett, Adeola Animasahun, Yvonne Bronner

**Affiliations:** School of Community Health and Policy, Morgan State University, Baltimore, MD USA

**Keywords:** Doulas, COVID-19, Breastfeeding support, Maternal and Child Health, Lactation Support, Birthing, Nursing Mothers, COVID-19 Guidelines

## Abstract

**Background:**

Doulas have been instrumental in providing breastfeeding support to nursing mothers before and during the COVID-19 pandemic, as they can significantly impact a mother's ability to initiate and maintain breastfeeding. However, the COVID-19 pandemic, subsequent lockdowns, and social isolation created challenges for nursing mothers to access doulas' services, usually provided in person. In this study, we examined the role of doulas in providing breastfeeding support during the COVID-19 pandemic, exploring adaptation to COVID-19 guidelines and the challenges doulas face in providing breastfeeding support during the pandemic.

**Methods:**

A systematic review was conducted following the PRISMA guidelines. Thirteen scientific databases and twenty peer-reviewed journals were searched for journal articles published in English between January 2020 and March 2022 using key search terms (e.g., Doula, Breastfeeding, COVID-19). Studies evaluating the role of doulas in providing breastfeeding support during COVID-19, and the impact of COVID-19 Guidelines on doula services, were included. Two reviewers independently performed the risk of bias assessment and data extraction. Summative content analysis was used to analyze the data.

**Results:**

The majority of studies were conducted in developed nations. This systematic review includes eight articles, four qualitative, one survey, two mixed-methods studies, and one prospective research study. Seven of the eight studies were conducted in the United States, and the eighth was conducted in multiple countries. These studies have three main themes: (1) virtual breastfeeding support provided by doulas during the pandemic; (2) remote social support provided by doulas to breastfeeding mothers during the pandemic; and (3) barriers to doula service delivery due to COVID-19 restrictions, primarily the exclusion of doulas as essential workers. The eight studies showed that doulas found innovative ways to serve the needs of birthing and nursing mothers during the difficulties brought on by the pandemic.

**Conclusion:**

Doulas provided breastfeeding support during the COVID-19 pandemic by utilizing innovative service delivery methods while navigating changes in COVID-19 guidance. However, system-level integration of doulas' work and the acknowledgment of doulas as essential healthcare providers are needed to enhance doula service delivery capacity, especially during a pandemic, to help improve maternal health outcomes.

## Background

The restrictions on non-essential gatherings, social distancing, and redirection of public health infrastructure were just a few of the diverse, wide-ranging strategies to mitigate and control the spread of COVID-19 around the globe [1]. At the onset of COVID-19, most healthcare systems prohibited "visitors and non-essential personnel" following guidelines from the Centers for Disease Control and Prevention (CDC) and other coordinating agencies. Doulas were a class of affected personnel deemed "non-essential" by health care systems and other health providers [2–5].

Doulas are birth workers who care for birthing people and their newborns—usually trained or certified community members who can serve and provide culturally appropriate maternal and reproductive health care [3, 6, 7]. They offer ongoing maternal health assistance to birthing people, including emotional, physical, and educational aid before, during, and after birth, supporting birthing people in navigating the health system and birthing process [3]. Their services are associated with positive maternal health outcomes, especially among low-income and marginalized racial and ethnic groups [8, 9]. Additionally, they can significantly impact a mother's ability to begin and sustain breastfeeding [10]. They provide breastfeeding support which can impact the continuity and duration of breastfeeding [4, 11].

### Breastfeeding support during the COVID-19 pandemic

The World Health Organization (WHO) recommends that newborns commence breastfeeding within an hour of birth, breastfeeding exclusively for the first half of their first year, and continue breastfeeding with the addition of complementary meals for at least two years [12]. This recommendation is not without challenges for nursing mothers, as they may encounter difficulties in infant latching, uncomfortable or painful nipples, and inadequate milk production, sometimes leading to the discontinuation of breastfeeding [13, 14]. Studies have shown that birthing people are frequently unprepared for the physical challenges of early breastfeeding, thus requiring breastfeeding support from certified lactation consultants and doulas who receive training in providing lactation support that can influence a mother's ability to initiate and continue breastfeeding [7, 15–17].

Breastfeeding support has inevitably changed due to COVID-19, with rates of exclusive breastfeeding among nursing mothers steadily declining [18, 19]. Delays in the early initiation of breastfeeding and inadequate breastfeeding support have contributed to this decline [18, 19]. Despite evidence that doulas can promote positive maternal and child health outcomes, the changing health system guidelines at the pandemic's peak deemed doulas non-essential. As a result, breastfeeding mothers had limited access to support, putting them at higher risk for postpartum anxiety, early termination of breastfeeding, and other health complications [4, 7, 20–22].

This systematic review examines the role of doulas in providing breastfeeding support during the COVID-19 pandemic and how community-based doula services adapted to changes in COVID-19 guidance to provide breastfeeding support during the pandemic.

## Methods

A systematic review was conducted in accordance with the PRISMA guidelines [23]. Thirteen scientific databases and twenty peer-reviewed journals were searched for journal articles published in English between January 2020 and March 2022 using key search terms (e.g., Doula, Breastfeeding, COVID-19). These databases were searched using the strategy outlined in the 2020 *Cochrane Handbook for Systematic Reviews of Interventions *[9]. There was no limit placed on the study design. (See Tables 1 & 2). Forty-three articles were identified; summative content analysis was used to analyze the data. A systematic review technique was chosen to comprehensively assess available literature, people, contexts, and the comparison of different kinds of articles.

**Table 1:** Sources: bibliographic databases and search engine

MEDLINE and MEDLINE In-Process & Other Non-Indexed Citations (Ovid)
Alt Health Watch
CINAHL (EBSCO)
Web of Science
EMBASE (Ovid)
PubMed.gov
PsycINFO (EBSCO)
Academic Search Complete (EBSCO)
Health Source
Academic Search Complete (EBSCO)
Library, Information Science & Technology Abstracts (LISTA)
Google Scholar

**Table 2:** List of journals searched

*American Journal of Public Health*
*Appetite*
*Birth Issues in Prenatal Care*
*BMC Pregnancy and Childbirth*
*Breastfeeding Medicine*
*Frontiers in Sociology*
*Health Communication*
*International Journal of Environmental Research and Public Health*
*Journal of Human Lactation*
*Journal of Obstetric, Gynecologic & Neonatal Nursing*
*Maternal & Child Nutrition*
*Maternal and Child Health Journal*
*Medical Anthropology*
*Midwifery*
*Pediatrics*
*PLoS One*
*The American Journal of Maternal/Child Nursing*
*The Journal of Perinatal Education*
*The Journal of Prenatal and Neonatal Nursing*

Google Scholar and the Web of Science were utilized to collect references and citation searches. The authors established current awareness alerts in databases such as MEDLINE, Embase, Web of Science, and Google Scholar to locate recent work. The authors conducted an iterative search to increase sensitivity, address indexing difficulties, and leveraged the Web of Science to find essential and influential electronic health journals. Manual searches were conducted by checking the online table of contents and using the journals' search engines (Table 1).

### Search strategies/search terms – the role of doulas in providing breastfeeding support during the COVID-19 pandemic

The search terms used included the following.

A. "doula" or "birth assistant" or "delivery companion" or "breastfeeding companion" or "childbirth companion" or "birth companion" or "birth partner" or "breastfeeding assistant" or "supportive companion" or "pregnant outreach" or "nursing companion" or "breastfeeding support" or "breastfeeding coach" or "all feeds."
B. "Breastfeed" or "human milk" or "infant nutrition" or "lactation" or "human lactation" or "breast milk" or "lactation" or "all feeds."
C. “Coronavirus” or “SARS-CoV-2” or “COVID-19” or “corona virus or “pandemic” or “coronavirus pandemic” or “SARS-CoV-12 pandemic or "all feeds."

### Data extraction and analysis—reference harvesting and citation searching

Authors obtained forty-three publications from 13 bibliographic databases, search engines, and manual searches. Following the database search, citations were added to Mendeley; duplicates were identified, eliminated, and then imported into Rayyan, where the remaining duplicates were deleted (Cochrane Collaboration, 2021). The Critical Appraisal Skills Program (CASP) approach for systematic reviews was utilized to evaluate the quality of the publications [24]. The titles and abstracts of the retrieved citations and papers deemed suitable were analyzed further using pre-defined inclusion and exclusion criteria. Preferred Reporting Items for Systematic Reviews and Meta-Analyses (PRISMA) were used to document reasons for exclusion [23].

### Inclusion and exclusion criteria

#### Inclusion criteria

Inclusion and exclusion criteria were developed using the study research questions. Studies that used qualitative methods (e.g., interviews, participant observation, focus group discussions) and quantitative studies, mixed-method studies, and randomized-controlled trials were included. Also, full-text articles in English published in peer-reviewed journals between January 2020 and March 2022 were included. Other inclusion criteria were:

- Papers that examined the role of community doulas in providing breastfeeding support to nursing mothers during the pandemic
- Papers that explored the role of community-based doula programs and delivery methods during the COVID-19 pandemic
- Papers that discussed how community-based doula services adapt to changes in COVID-19 guidance to provide breastfeeding support during the pandemic.
- Papers that explored how community-based doula care has impacted maternal health and service delivery during the COVID-19 pandemic.

#### Study exclusion criteria

Articles excluded were:

- Reviews
- Secondary analyses (reviews, previews, opinions, editorials, case studies, protocols, conference abstracts, and news articles)

In addition, papers in languages other than English, articles without full-text, papers that scored eight or less using the CASP tool, those not directly related to the topic or population of interest, and research conducted before January 2020 were excluded.

## Results

**Fig. 1:**
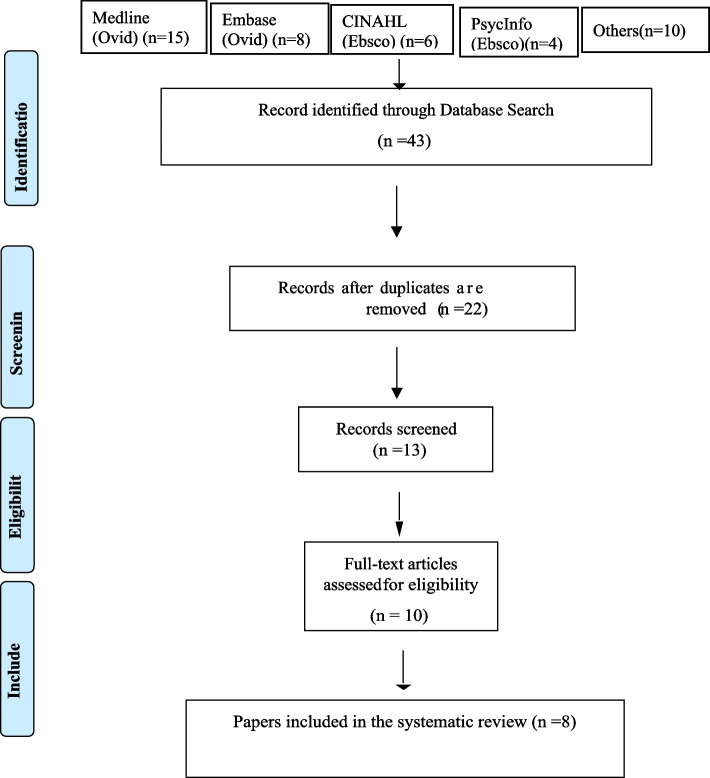
PRISMA Flow Chat

The eight articles in this systematic review include four qualitative, one survey, two mixed-methods, and one prospective research study. Seven of the eight were conducted in the United States, and the eighth was conducted in multiple countries. Participants in these studies were birth workers, including doulas. The search identified forty-three publications: 15 in MEDLINE, 8 in EMBASE, 6 in CINAHL, 4 in PsycINFO, and 10 in the other databases (see Fig. 1). Deduplication and filtering decreased the number of documents to 22, which were evaluated for eligibility using the full text. After eligibility screening, the number of articles was reduced to 10, and these were critically reviewed using the Critical Appraisal Skills Program (CASP). Two reviewers (MO and AA) separately assigned a score of 10 to each paper by answering "yes" to all ten elements on the CASP checklist. Eight articles had a perfect inter-rater agreement (An overall understanding of 98 percent). A third reviewer (SB) resolved disagreements. On a scale from 1 to 10, two papers received a score of 9, and six papers received a score of 10; these eight were the final papers included in the study (see Table 3 and Fig. 1).

**Table 3:** Studies presenting results on the role of doulas in providing breastfeeding support during the COVID-19 pandemic

Reference	Journal	Study Design	Study Population	Result
Adams, 2021 (United States) [25]	*Birth*	Qualitative research: Semi-structured phone interviews with 15 birth doulas to discuss COVID-19-related pregnancy and birth challenges and how doulas navigated these challenges	*N* = 15 Doulas	Doulas clients experience three primary pregnancy and childbirth barriers related to COVID-19: (i) fear of exposure, (ii) limited access to their intended support systems, and (iii) uncertainty about hospital restrictions on labor and delivery. Doulas responded deftly, helping their clients face these obstacles
Nguyen et al., 2021 (United States) [26]	*Health Communication*	Qualitative research: Thematic analysis of 25 semi-structured interviews of doulas in the United States to understand the impact of the pandemic on doula service delivery to birthing people	*N* = 25 Doulas	Despite the difficulties in providing remote services for clients during the pandemic, telemedicine gave doulas a way to connect with them and teach them coping mechanisms. Doulas' experiences during the pandemic may shape future care practices, especially for low-income and underserved communities
Oparah et al., 2021 (United States) [27]	*Frontiers in Sociology*	Qualitative research: Narratives from Black birth workers to investigate the effects of hospital and clinic restriction on maternal and child health standards of care as well as the presence of birth support staff and doulas during the pandemic	*N* = 38 birth health workers, including doulas*,*	Black doulas navigated racial tensions and violence to support nursing mothers during the pandemic
Reyes, 2021 (United States) [28]	*Frontiers in Sociology*	Mixed methods: Assess the impact of COVID-19 on childbirth in Puerto Rico	*N* = 11 birth health workers, including doulas	The lack of institutional support for Puerto Rican midwives and doulas poses significant challenges to doula services during the pandemic
Rivera, 2021 (United States) [29]	*Frontiers in Sociology*	Mixed methods: Documentation of community-based doulas' transition to virtual work with Black and Afro-Latinx communities, beginning in 2019 through the COVID-19 pandemic, using online interviews and virtual meetings to document the transition from face-face to virtual encounters	*N* = 6 Doulas	Despite various barriers encountered by doulas in New York State, two organizations in this study provided tailored maternal health support to black and brown mothers during the pandemic
Schindler-Ruwisch & Phillips, 2021 (United States) [13]	*Journal Of Human Lactation*	Prospective study: An online survey using the Qualtrics platform to determine changes in breastfeeding support services reported by certified lactation providers during the COVID-19 pandemic	*N* = 39 Certified lactation providers, including doulas	Although virtual breastfeeding support was beneficial during the pandemic, visits to lactation professionals plummeted. Technical, logistical, and difficulty assisting with latching, among others, were significant challenges that doulas and their clients encountered
Searcy & Castañeda, 2021 (Multiple countries) [30]	*Frontiers in Sociology*	Qualitative: Interviews of 515 doulas in 24 countries to learn about their experiences navigating service delivery during the COVID-19 pandemic. Seventy-five percent of respondents were doulas in 42 of the 50 states in the US. The remaining participants are from 22 countries, including South Africa, Canada, Australia, Germany, Italy, Finland, Portugal, Ireland, Sweden, Japan, the United Kingdom, Taiwan, Israel, Peru, India, Dubai, Mexico, Argentina, and Bolivia	*N* = 515 Doulas	A key theme of this study is the absence of a doula during births and the accompanying necessity to help birthing people virtually. Doulas also had mixed emotions about the efficacy of virtual support, raising concerns about COVID's long-term effects on their profession
Davis-Floyd et al., 2020 (United States) [31]	*Medical Anthropology*	Survey: Online survey to assess the effects of the changing COVID-19 guidelines on birthing options during the COVID-19 pandemic and the future policy implications of those guidelines to improve maternal care during a pandemic and any other crisis	*N* = 41 Health workers, including doulas	Doulas were barred from birthing rooms, leaving mothers alone; vagueness in COVID-19 guidelines made it difficult for providers, including doulas

### Doulas providing breastfeeding and maternal health support during the COVID-19 pandemic

Although doula care has numerous advantages, accessing these services is not without difficulties, some of which have grown more restrictive since the COVID-19 Pandemic [32].To combat the spread of COVID-19, hospital systems implemented substantial modifications to their policies and standards at the start of the pandemic, including restricting visits and doula services [33, 34]. Evolving guidelines, uncertainty, and subsequent mitigation strategies altered mothers' expected birth location and nursing intentions [30, 35, 36].This situation was further exasperated due to a lack of appropriate legislation or hospital policies to acknowledge the vital work of doulas: they were deemed non-essential and largely unable to participate in births, even though expecting parents had come to rely on doula support [3, 32, 37]. Additionally, Doulas struggled to transition to virtual services during this time to deliver much-needed services. Home visits, a vital part of doula services, were also curtailed during the pandemic. These challenges underscore the lack of support, collaboration, and integration into the healthcare system, problems that doulas face even when there is no pandemic [3, 32].

The results from this systematic review emphasized the significance of breastfeeding support provided by doulas and its potential. The eight studies demonstrated that doulas found innovative ways to serve the needs of birthing and nursing mothers during the difficulties brought on by the pandemic, whether through virtual breastfeeding support, delivering essential supplies to clients, navigating racial tensions, or providing educational and mental health support [5, 13, 25, 27–29, 31].

A prospective, anonymous online survey of Gatekeepers at Connecticut agencies and breastfeeding networks of eligible lactation personnel reported associated changes in lactation services, even though breastfeeding continued throughout the epidemic. The authors concluded that breastfeeding inequities might be further exacerbated without fair access to lactation support, suggesting that future adaptive alternatives in the sector might be impacted by the difficulties and breakthroughs in virtual assistance [13]. There was a need for breastfeeding support and the potential benefits of enhanced mother-infant interaction during the lockdown period. Breastfeeding disparities might worsen for people who don't have access to lactation support. These individuals need in-person or virtual support to mitigate lactation-associated challenges [13]. This study reinforces the need to conduct additional research on the impact of the COVID-19 breastfeeding guidelines on doulas' support services during the pandemic in preparation for any future epidemic or pandemic.

### Impact of COVID-19 guidelines on doula services

Studies that evaluated the impact of COVID-19 guidelines on doula services found that doulas clients experienced three primary pregnancy and childbirth barriers: (i) fear of exposure, (ii) limited access to their intended support systems, including doulas, and (iii) uncertainty about hospital restrictions and COVID guidelines on labor and delivery [25, 26, 31]. Doulas responded deftly, helping their clients face these obstacles. Studies demonstrate that doulas were willing to go the extra mile to help birthing people and nursing mothers navigate COVID-19-related barriers [25]. Despite the difficulties in providing remote services for clients during the pandemic, telemedicine gave doulas a way to connect with their clients and teach them coping mechanisms. These experiences of doulas during the pandemic may shape future care practices, especially for low-income and underserved communities [26]. However, doulas expressed significant concerns that their classification as "non-essential" employees continued even after COVID-19 restrictions were eased. Most doulas still could not attend births—limiting their ability to provide needed services like emotional and breastfeeding support to new mothers. Doulas had mixed emotions about virtual assistance effectiveness [26, 37]. They were concerned about COVID's long-term effects on their profession and worried about maltreatment and obstetric violence as birthing people enter hospitals unaccompanied. For various reasons, doulas prefer to work with their clients in person rather than virtually [37]. In evaluating the effect of the exclusion of doulas from delivery rooms due to COVID-19 restrictions, a shift in birth plans was noted by birthing people from hospital to home births where they could have doulas and partners present [31]. During the peak of the pandemic, most people hiring doulas were wealthy and educated. Low-income and underserved birthing people could not afford this benefit and were limited to the services that doulas could provide [26, 31]. This only increased the existing access inequalities that low-income and underserved birthing people face. Doulas were able to provide families with birth information and virtually support their clients during labor. The exclusion of doulas also critically impacted hospital staff, who expressed dismay that they could not provide women in labor and postpartum with tailored support during this period; in this situation, the presence of doulas in the hospital would have been beneficial [26, 38].

### Adoption of COVID_19 guidelines and modification of service delivery methods

Two studies included in this review examined the impact of hospital and clinic restrictions, including restrictions on the attendance of birthing support staff, including doulas. Oparah collected narratives of Black and Latinx birth workers [27, 29]. In these studies, doulas devised innovative ways to deal with COVID-19 restrictions. They reported offering more prenatal and extended postpartum visits and maintained contact with their clients virtually and through telecommunication [27]. Many birth workers empowered their clients to self-advocate and take greater responsibility for their prenatal care. Doulas expanded their scope of work by helping with grocery shopping and other maternal health supports [27].To close the health disparity gaps, doulas helped their clients figure out telemedicine, especially for those who are not internet savvy or lack resources that can support virtual doula services. Black birth workers used a holistic approach to care during the pandemic, finding creative methods to establish a community that supported new parents. These approaches were key in addressing US maternal and child health disparities [27, 32].

In examining Black and LatinX community-based doula work transitions in the United States during the Pandemic, Rivera (2021) found that doulas had to adapt to new ways of delivering services to keep themselves, their clients, and the community safe. Doulas reached their community and birthing clients through free virtual childbirth courses, including breastfeeding support [29].In an investigation of how COVID-19 affected births in Puerto Rico and how the pandemic differed or is similar to past disasters, a study included in this review interviewed eleven Puerto Rican women working in reproductive health and justice. This study found that Puerto Rican midwives and doulas lacked institutional support despite the growing popularity of doula services and midwife-attended births. In Puerto Rico, the lack of legal recognition and rights for homebirth midwives and doulas is a severe concern. This suggests the need for legal recognition and the promotion of the rights of doulas [28].

## Discussion

This review presented evidence that doulas played crucial roles in providing breastfeeding and other maternal health support to birthing people throughout the pandemic while utilizing innovative ways to adapt to COVID-19 guidelines and health system policies. Common themes in the literature were that the COVID-19 guidelines and health system policies relegating doulas to non-essential personnel substantially impacted their capacity to adequately assist their clients during the pandemic, especially in the areas of breastfeeding and emotional support [5, 13, 25, 27]. Because their clients are vulnerable to the possibility of experiencing medical racism and discrimination, doulas who work primarily with birthing people of color often take on advocacy as a significant part of their work. However, with the shift to virtual care, the physical presence that doulas see as a crucial part of their advocacy is lost. Additionally, doulas encounter difficulties in helping their clients switch to virtual support [27]. They note that breastfeeding disparities might worsen for those without equitable access to lactation support. Doulas also have mixed emotions about the efficacy of virtual support, raising concerns about COVID's long-term effects on their profession [13, 25]. Despite these challenges, doulas maintained the personal nature of their work. They provided more in-depth informational support during the prenatal period to increase clients' and partners' self-efficacy and incorporated visual aids. Most notably, doulas coached partners during COVID-19 to carry out tasks that doulas would typically do, such as offering physical interventions and providing emotional support to the birthing person [13, 25].

### Policy and practice implications

Doula activities during the COVID-19 pandemic have significant policy implications that can affect the workforce pathway and indicate ways the workforce could be improved or further mobilized. Given the proper recognition and support, doulas and birth workers can provide more appropriate services to pregnant and nursing mothers.

Areas for consideration include:

1. Increasing access to high-quality care for all people giving birth, especially in underserved communities, through integrating doulas into the health system
2. Recognizing birth doulas as essential care team members in hospitals and other healthcare facilities and insurance companies covering doulas' services
3. Using the same guidelines for doulas and birthing workers as those used for other medical professionals during the pandemic
4. Supporting laws to enhance the sustainability of care models that utilize community-based doulas.

Most articles in this systematic review focus on US doula activities during the COVID-19 pandemic. These recommendations reflect policies in the United States but may be adapted for use by doulas and policymakers worldwide.

The studies included in this systematic review have demonstrated the value of doula care during the pandemic regardless of the COVID-19 guideline restrictions. Doula care can help close inequity gaps by improving access to patient-centered, culturally appropriate, customized treatment and advocacy in medical environments where such investments may not be present. Integration of doulas into collaborative maternity approaches strengthens the care team's response to birthing people.

Future research should examine the work of doulas during the pandemic and studies that provide baseline data for advocacy policy implementation that will fully integrate doulas as essential to the healthcare workforce. Research should also focus on the perspectives of birthing people at risk for adverse maternal health outcomes, informing how doula care can help mitigate health disparities. It will also be vital to examine how doula services can be fully integrated into the health system and how that may correlate with health outcomes for birthing people and families, especially during a pandemic. Although there is preliminary evidence of the modifications made to the lactation field in response to COVID-19, more research into these modifications and their long-term effects is necessary.

### Study limitation

A significant limitation of this review is that most of the articles were studies conducted in the United States. It does not offer a comprehensive view of doulas in other countries and their approaches to providing care during a pandemic. Additionally, this review relied on the critical appraisal of individual studies, and the interpretation of results in each paper examined. Despite these limitations, this study contributes to the larger body of literature on doula care approaches during the COVID-19 pandemic by highlighting their role in supporting the maternal and child health continuum in emergency and non-emergency situations. Additionally, this review supports the calls for system-level integration of doulas' work and the need to recognize doulas as crucial healthcare professionals who can contribute to improving maternal health outcomes.

## Conclusion

Doulas provide their clients with vital maternal health services despite the hurdles presented by the COVID-19 pandemic. They use innovative service delivery methods to offer breastfeeding support while navigating changes in COVID-19 guidance. By deeming them non-essential and disregarding their role in advancing maternal health services during the pandemic, the healthcare system may have missed an opportunity to integrate a dependable workforce into the maternal health continuum that can assist in improving health outcomes for mothers and children. System-level integration of doulas' work and the acknowledgment of doulas as essential health care providers are needed to enhance doula service delivery capacity, especially during a pandemic, to sustain positive maternal health outcomes.

## Data Availability

The data used and analyzed during this study are available from the corresponding author upon reasonable request.
